# Functional Group-Dependent Induction of Astrocytogenesis and Neurogenesis by Flavone Derivatives

**DOI:** 10.3390/biom9120812

**Published:** 2019-12-02

**Authors:** Ha-Rim Lee, Jin Mi Kang, Young Min Kim, Sagang Kim, Jihyae Ann, Jeewoo Lee, Hyun-Jung Kim

**Affiliations:** 1 College of Pharmacy, Chung-Ang University, Seoul 06974, Korea; harim9201@hanmail.net (H.-R.L.); cholove_00@naver.com (Y.M.K.); 2 College of Pharmacy, Seoul National University, Seoul 08826, Korea; jinmi98@snu.ac.kr (J.M.K.); carolroom12@naver.com (S.K.); jihuya@gmail.com (J.A.)

**Keywords:** astrocytogenesis, neurogenesis, neural stem cells, differentiation, flavone, structure-activity relationships

## Abstract

Neural stem cells (NSCs) differentiate into multiple cell types, including neurons, astrocytes, and oligodendrocytes, and provide an excellent platform to screen drugs against neurodegenerative diseases. Flavonoids exert a wide range of biological functions on several cell types and affect the fate of NSCs. In the present study, we investigated whether the structure-activity relationships of flavone derivatives influence NSC differentiation. As previously reported, we observed that PD98059 (2′-amino-3′-methoxy-flavone), compound **2** (3′-methoxy-flavone) induced astrocytogenesis. In the present study, we showed that compound **3** (2′-hydroxy-3′-methoxy-flavone), containing a 3′-methoxy group, and a non-bulky group at C2′ and C4′, induced astrocytogenesis through JAK-STAT3 signaling pathway. However, compound **1** and **7–12** without the methoxy group did not show such effects. Interestingly, the compounds **4** (2′,3′-dimethoxyflavone), **5** (2′-*N*-phenylacetamido-3′-methoxy-flavone), and **6** (3′,4′-dimethoxyflavone) containing 3′-methoxy could not promote astrocytic differentiation, suggesting that both the methoxy groups at C3′ and non-bulky group at C2′ and C4′ are required for the induction of astrocytogenesis. Notably, compound **6** promoted neuronal differentiation, whereas its 4′-demethoxylated analog, compound **2**, repressed neurogenesis, suggesting an essential role of the methoxy group at C4′ in neurogenesis. These findings revealed that subtle structural changes of flavone derivatives have pronounced effects on NSC differentiation and can guide to design and develop novel flavone chemicals targeting NSCs fate regulation.

## 1. Introduction

Neural stem cells (NSCs) are self-renewing and multipotent cells present in both embryonic and adult brain [1,2,3], which can generate neurons, astrocytes, and oligodendrocytes [3]. Endogenous NSCs are used for cell therapy due to their capacity to differentiate and restore the loss of neurons [2,3,4,5]. The fate of NSC is regulated by extrinsic factors and the culture environments in addition to intrinsic mechanisms [6,7,8,9,10,11,12,13,14]. For example, intrinsic factors such as the basic helix–loop–helix proteins, Mash1, and Neurogenin2, are involved in the acquisition of neuronal cell fate [15,16,17]. It has been known that truncated tropomyosin receptor kinase B, a receptor for brain-derived neurotrophic factor, directs NSCs to glial cell fate [18]. NSCs respond to and are regulated by small molecules to change the fate of NSCs [7,8,9,14,19,20,21,22,23,24].

Flavonoids are found in plants and exert a wide range of pharmaceutical activities, such as anti-inflammatory, anti-oxidant, and anti-tumor activities [25,26,27]. Recent studies have reported the neuroprotective and neurogenic properties of flavonoids [28,29,30,31,32,33,34,35]. Of these, the component of *Ginkgo biloba* leaf extract, kaempferol (3,4′,5,7-tetrahydroxy-flavone), inhibits rat brain monoamine oxidase A and B and protects against *N*-methyl-d-aspartate-induced neurotoxicity in rat neuronal cultures [31]. Similarly, Wogonin (5,7-dihydroxy-8-methoxy-flavone) isolated from *Scutellaria baicalensis* root promotes neuronal differentiation of the hippocampal stem cell line HiB5 and induces neurite outgrowth of primarily cultured rat cortical NSCs [29]. In addition, several studies have revealed that intake of flavonoids correlates with better cognitive performance and is inversely related to the risk of dementia [36,37].

The structural diversity of flavonoids or flavone derivatives provides a distinct biological process. For example, the radical scavenging is related to the *o*-dihydroxy group in the B-ring, the C2–C3 double bond with a 4-oxo function, and the 3- and 5-hydroxy group with a 4-oxo function [38,39]. The chemical structures essential for neuroprotection are the hydroxyl moieties at C3, C5, and C7 [38,40]. We have recently demonstrated that mitogen-activated protein kinase (MAPK)/extracellular signal-regulated kinase (ERK) kinase (MEK) inhibitor and a flavone derivative, PD98059, have induced astrocytogenesis through Janus kinase (JAK)-STAT3 signaling activation [41]. In addition, we have demonstrated that the deaminated PD98059 (3′-methoxy-flavone or compound **2**), however, promotes astrocytic differentiation without inhibiting ERK1/2 activation, which suggests that MEK inhibition is not necessary for the astrocytogenesis [41]. These findings suggest that the chemical structure of flavone derivatives plays critical roles in NSC fate determination.

In this study, we aimed to discover new flavone molecules that can regulate NSC fate to develop new therapeutic agents for neurodegenerative diseases. Therefore, we investigate the effects of ten additional flavone derivatives (compound **3**–**12**) with PD98059, compound 2, and flavone on rat NSC differentiation. Furthermore, we determined structural features essential for the regulation of NSC differentiation.

## 2. Methods

### 2.1. Chemistry

Flash column chromatography was performed using silica gel 60 Å, 230−400 mesh (Merck Millipore, Burlington, MA, USA) with the indicated solvents. ^1^H NMR spectra were recorded on a JEOL JNM-LA300 instrument at 300 MHz. Chemical shifts were reported in ppm with tetramethylsilane (Me4Si) as a reference standard. Mass spectra were recorded on a VG Trio-2 GC-MS system. Detailed synthetic procedures and characterization data of final compounds are presented in the Supporting Information.

### 2.2. NSC Culture

NSCs were isolated from E14 Sprague-Dawley rat cortex (Orient Bio, Seongnam, Republic of Korea) and expanded as a neurosphere in Dulbecco’s modified Eagle medium/F12 supplemented with 1% (*v*/*v*) antibiotic-antimycotic (Thermo Fisher Scientific, Waltham, MA, USA), 2% (*v*/*v*) B27 (Thermo Fisher Scientific), and 20 ng/mL each of EGF and FGF2 (Merck Millipore). After 6 days, the cells were dissociated, plated onto 0.01% poly-d-lysine (Sigma-Aldrich, St. Louis, MO, USA) and 10 μg/mL laminin (Thermo Fisher Scientific) coated plates and expanded for 1 day. The final differentiation step involved removing EGF/FGF2 and treating with 0.1% DMSO (Sigma-Aldrich), 20 μM PD98059 (Merck Millipore), or 20 μM the synthesized compounds (Table 1) after 1 h, 3 days, or 4 days. Animal experiments were performed following Chung-Ang University and NIH standards of animal care.

### 2.3. Immunocytochemistry

After 4 days of treatment, the cells were fixed in 4% paraformaldehyde (Biosesang, Seongnam, Republic of Korea) and rinsed with phosphate-buffered saline (PBS). Subsequently, the fixed cells were blocked in 5% normal goat serum (Merck Millipore) supplemented with 0.2% Triton X-100 (VWR International, Radnor, PA, USA) in PBS and incubated with primary antibodies such as anti-glial fibrillary acidic protein (GFAP; rabbit IgG, 1:1000; Agilent, Santa Clara, CA, USA) or TuJ1 (mouse IgG2b, 1:1000; Sigma-Aldrich). Then the cells were rinsed in PBS and incubated with secondary antibodies conjugated to Cy3 (goat anti-rabbit IgG, 1:1000; Jackson ImmunoResearch, West Grove, PA, USA) or Alexa Fluor 488 (goat anti-mouse IgG, 1:1000; Thermo Fisher Scientific). Total number of cells was counted by DAPI (1:10,000 in PBS; Sigma-Aldrich) staining. Images were obtained with an inverted fluorescence microscope (DMIL; Leica, Wetzlar, Germany).

### 2.4. Real-Time RT-PCR

Total RNA was isolated from cells using TRIzol reagent (Thermo Fisher Scientific). Reverse transcriptase-polymerase chain reaction (RT-PCR) was carried out using the QuantiTect Reverse Transcription Kit (Qiagen, Hilden, Germany) following the manufacturer’s protocol to prepare first-strand complementary DNA (cDNA). RT-PCR was performed using the iQ SYBR Green Supermix (Bio-Rad, Hercules, CA, USA). The following primer sets (Cosmo Genetech, Seoul, Republic of Korea) were used to amplify cDNA: *βIII tubulin*, agccctctacgacatctgct (forward) and attgagctgaccagggaatc (reverse); *gfap*, agcggctctgagagagattc (forward) and agcaacgtctgtgaggtctg (reverse); or *gapdh*, agttcaacggcacagtcaag (forward) and gtggtgaagacgccagtaga (reverse). The PCR conditions were as follows: initial activation at 95 °C for 3 min followed by 40 cycles of denaturation at 95 °C for 10 s, annealing at 58 °C for 15 s and extension at 72 °C for 20 s. The housekeeping gene *gapdh* was used as an internal control.

### 2.5. Western Blot Analysis

Cells were washed with PBS and lysed in NP-40 lysis buffer. The lysates were centrifuged at 25,200× *g* for 20 min to remove debris. The proteins were denatured by boiling for 5 min in sodium dodecyl sulfate (SDS) sample buffer, loaded onto SDS-polyacrylamide gel, separated electrophoretically, and transferred to polyvinylidene fluoride membrane (Merck Millipore). The membranes were incubated with 5% skim milk or bovine serum albumin (Merck Millipore) in 20 mM Tris-buffered saline containing 0.03%–0.1% Tween 20 (VWR International) to block non-specific protein binding. The blots were probed with primary antibodies; anti-GFAP (1:500), TuJ1 (1:2000), GAPDH (1:1000, Santa Cruz, Dallas, TX, USA), anti-phospho-STAT3 (Tyr705, 1:2000, Cell Signaling, Danvers, MA, USA), anti-STAT3 (1:2000, Cell Signaling), anti-phospho-ERK1/2 (Thr202/Tyr204, 1:4000, Cell Signaling), and anti-ERK1/2 (1:4000, Cell Signaling, Danvers, MA, USA) followed by horseradish peroxidase-conjugated secondary antibodies; anti-rabbit IgG (1:5000) or anti-mouse IgG (1:5000, Santa Cruz). The protein bands were visualized using Western Blotting Luminol Reagent (Santa Cruz).

### 2.6. Statistical Analysis

Values were expressed as means ± standard error of the mean (SEM), and statistical significance was determined using Student’s *t*-test (* p < 0.05, ** p < 0.01).

## 3. Results and Discussion

### 3.1. Design and Synthesis of Flavone Derivatives

**Scheme 1:**
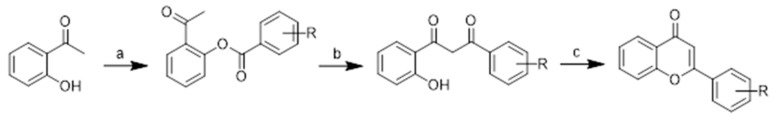
General synthesis of flavone derivatives. Reagents and conditions: (a); (i) ArCO_2_H, (COCl)_2_, DMF, (ii) anhydrous pyridine, r.t, 3 h; (b) KOH pyridine, 50 °C, 30 min; and (c) H_2_SO_4_, AcOH, reflux, 1 h.

A series of flavone derivatives with various substitutions on the B-ring were designed and synthesized to investigate the structural requirements of flavone derivatives that can modulate the fate of NSC. The derivatives were synthesized by the method of the Allan-Robinson reaction with the corresponding benzoyl chloride (Scheme 1). The synthesized compounds are listed in Table 1, along with flavone and PD98059.

### 3.2. The 3′-Methoxy Group (Methoxy Group at R_2_ Position) and Non-Bulky Group at C2′ (R_1_) and C4′ (R_3_) on Flavone Are Essential for Induction of Astrocytogenesis

**Figure 1:**
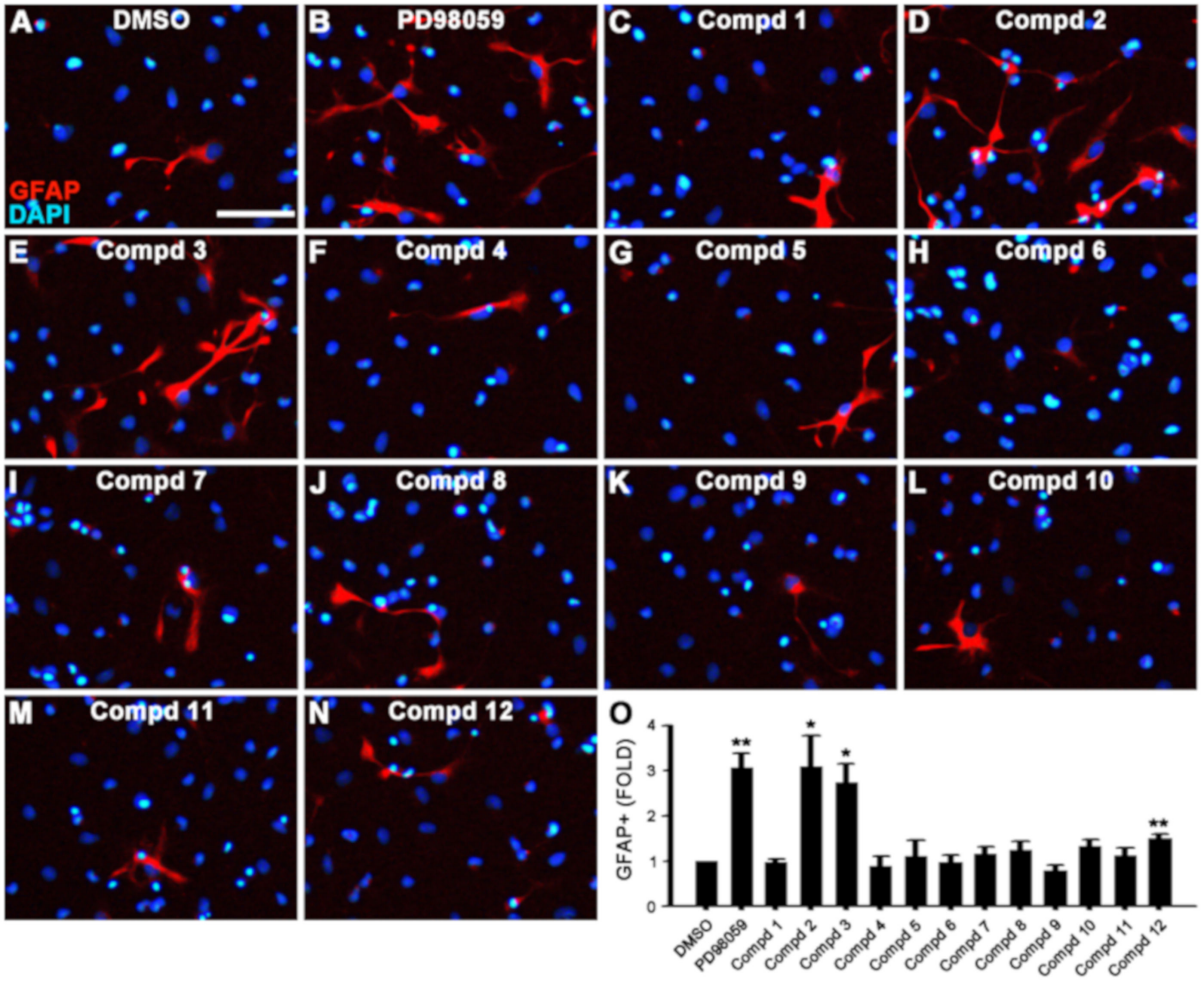
PD98059, compound **2** and **3** increased the differentiation of neural stem cells (NSCs) into anti-glial fibrillary acidic protein (GFAP)-positive astrocytes. (**A–N**) NSCs were treated with 0.1% DMSO or 20 μM of each compound, fixed after 4 days, stained with anti-GFAP (red), and counterstained with DAPI (blue). Scale bar = 50 μm. (**O**) Quantification of GFAP-positive cells among total DAPI-positive cells. Data are shown as mean fold change ± SEM of three independent experiments. * p < 0.05, ** p < 0.01 (Student’s *t*-test).

**Figure 2:**
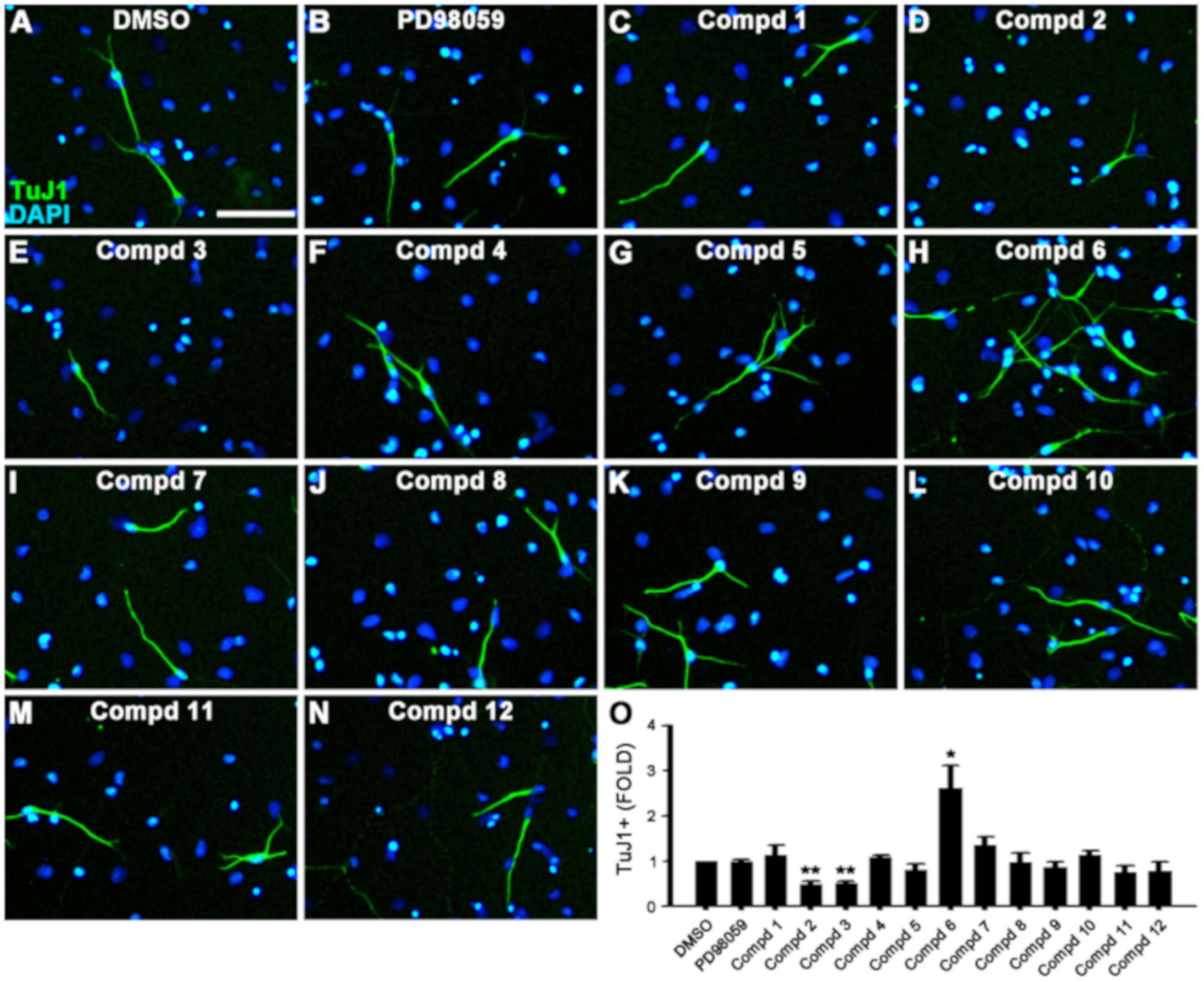
Compound **6** increased the differentiation of NSCs into TuJ1-positive neurons. (**A–N**) NSCs were treated with 0.1% DMSO or 20 μM of each compound, fixed after 4 days, stained with TuJ1 (green), and counterstained with DAPI (blue). Scale bar = 50 μm. (**O**) Quantification of TuJ1-positive cells among total DAPI-positive cells. Data are shown as mean fold change ± SEM of three independent experiments. * p < 0.05, ** p < 0.01 (Student’s *t*-test).

**Figure 3:**
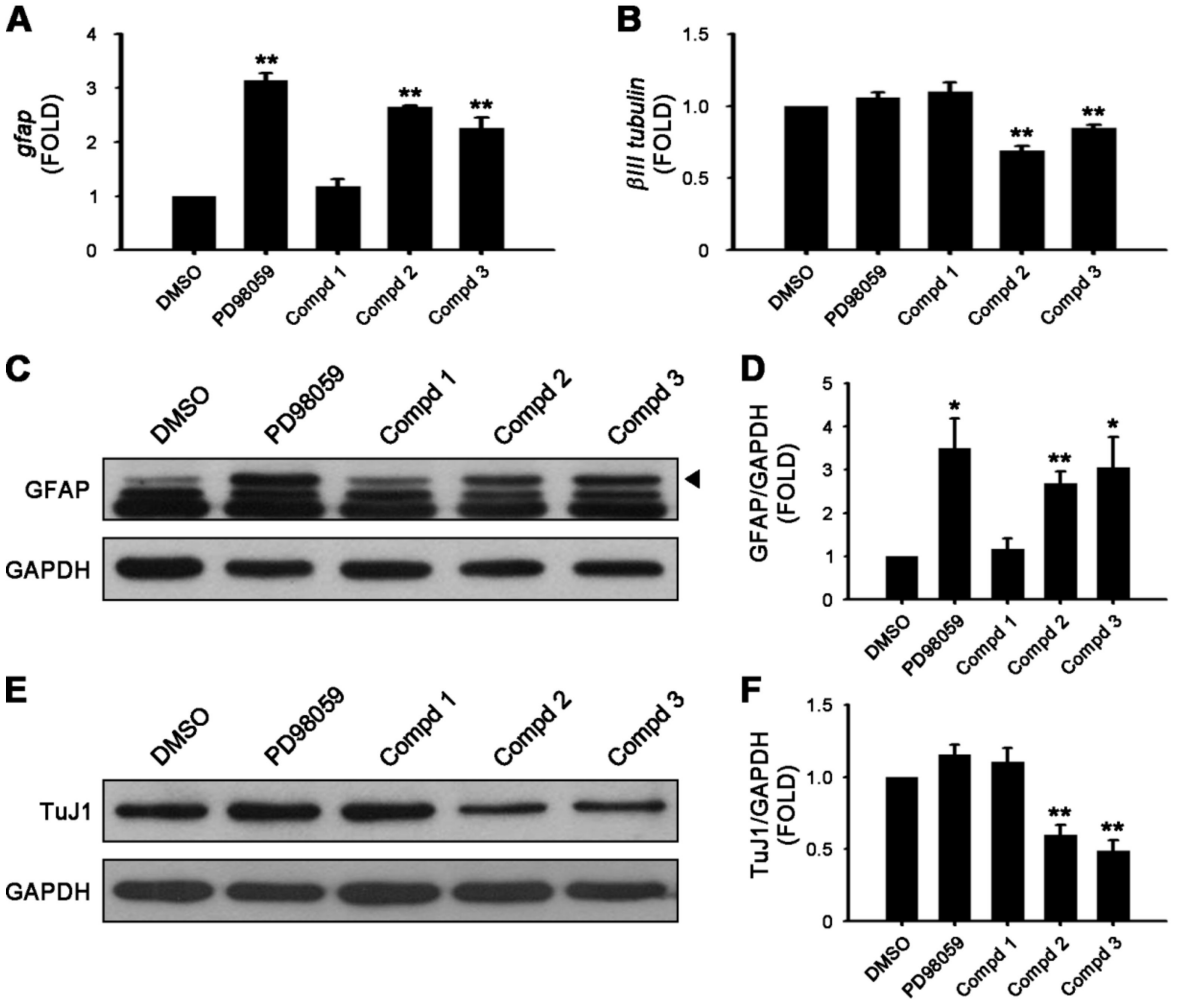
PD98059, compound **2,** and **3** induced GFAP mRNA and protein levels. (**A**,**B**) After treatment of NSCs with vehicle or each compound for 3 days, total RNA was isolated and used to perform quantitative RT-PCR to quantify (**A**) *gfap* and (**B**) *βIII tubulin* mRNA levels. Data are shown as mean fold change ± SEM of three independent experiments. (**C**–**F**) Levels of (**C**,**D**) GFAP and (**E**,**F**) βIII Tubulin protein were determined by western blot analysis after 4 days of the treatment with the selected compounds. GAPDH was used as a loading control. A representative immunoblot and quantification of three separate experiments are shown. * p < 0.05, ** p < 0.01 (Student’s *t*-test).

**Figure 4:**
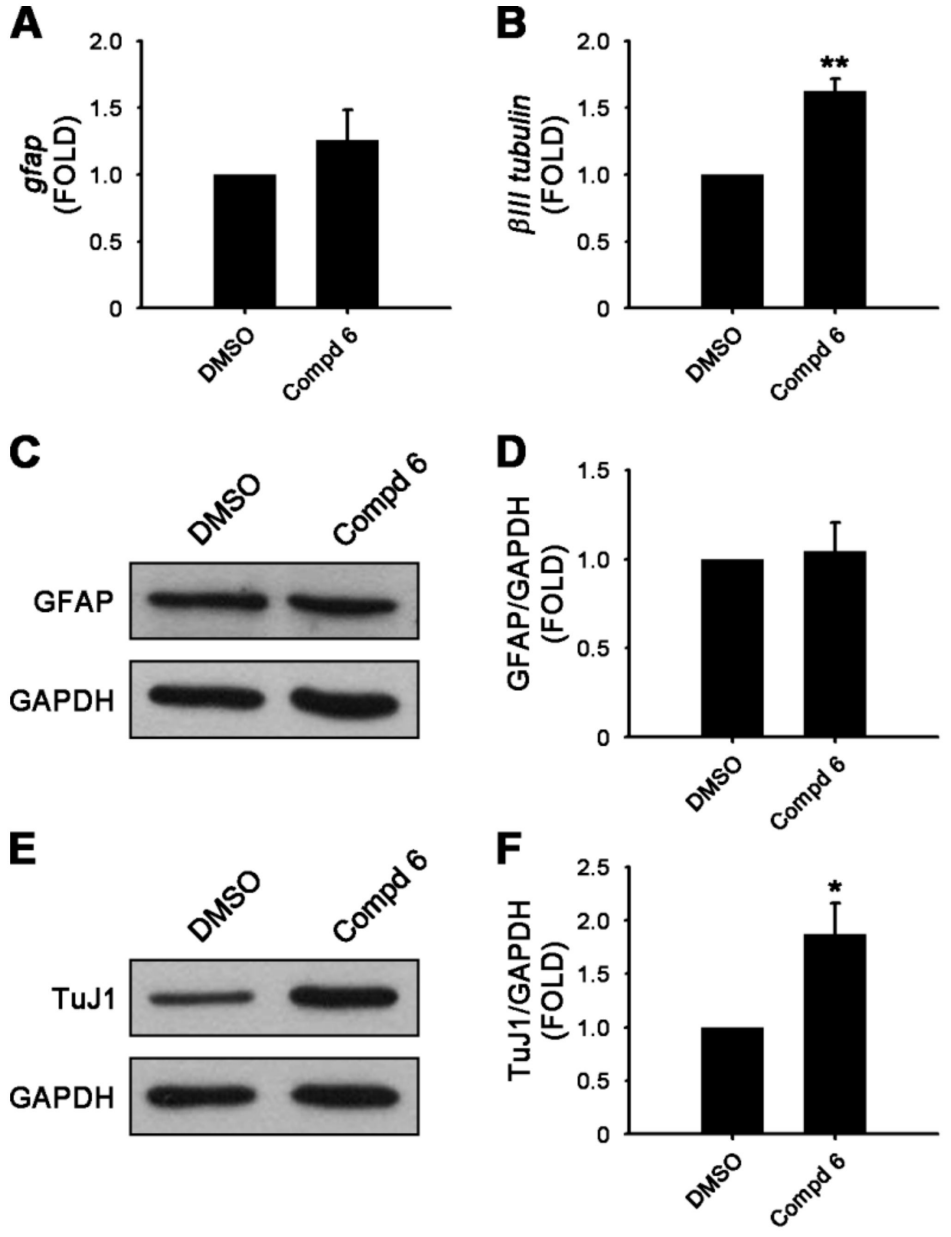
Compound **6** induced βIII tubulin mRNA and βIII Tubulin protein levels. (**A**,**B**) After treatment of NSCs with vehicle or each compound for 4 days, total RNA was isolated and used to perform quantitative RT-PCR to quantify (**A**) *gfap* and (**B**) *βIII tubulin* mRNA levels. Data are shown as mean fold change ± SEM of three independent experiments. (**C–F**) Levels of (**C**,**D**) GFAP and (**E**,**F**) βIII Tubulin protein were determined by western blot analysis after indicated treatment for 4 days. GAPDH was used as a loading control. A representative immunoblot and quantification of three separate experiments are shown. * p < 0.05, ** p < 0.01 (Student’s *t*-test).

We explored the effects of various flavone derivatives (Table 1) on the differentiation of NSCs (Figure 1, Figure 2, Figure 3 and Figure 4). Immunocytochemistry was performed using anti-GFAP to detect differentiated astrocytes. For the purpose, we used PD98059 and compound **2** as the positive controls and flavone as the negative control to measure the effect of different flavone derivatives in the induction of astrocytogenesis [41]. Among the tested flavone derivatives, PD98059 compounds **2** and **3** significantly increased the number of GFAP-positive astrocytes compared to dimethyl sulfoxide (DMSO)-treated control (Figure 1). The results were confirmed by RT-PCR followed by real-time PCR and western blot analysis. It was observed that PD98059, compounds **2**, and **3** increased the levels of both GFAP mRNA and protein (Figure 3A,C,D).

The results described above showed that only three out of 13 flavone derivatives increased astrocytogenesis (PD98059, compound **2**, and **3**), suggesting that specific structural features were related to astrocytogenic activity. These flavone derivatives have the 3′-methoxy group in R_2_ position (Table 1) in common. In contrast, the flavone derivatives lacking 3′-methoxy group (compounds **1, 7**–**12**), showed no astrocyte inducing activity (Figure 1). However, it is interesting to note that compound **4**–**6**, with 3′-methoxy moiety in R_2_ position, also did not show astrocytogenic activity (Figure 1). When NH_2_, H, or OH is substituted by bulkier groups like OCH_3_ or NHCOCH_2_Ph at C2′ (R_1_ position), the activity was lost, as in compound **4** and **5** (Figure 1). The methoxylation at C4′ (R_3_ position) caused a loss of activity potential, as seen with compound **6** (4′-methoxylated compound **2**; Figure 1). 

The results of the present study suggested that the structural requirements for astrocytogenesis include not only the methoxy group at C3′ (R_2_ position) but also a non-bulky group at C2′ (R_1_ position) and C4′ (R_1_ position). Similar to our results that flavone derivatives, compound **2** and **3**, induce astrocytogenesis, various flavonoids are reported to influence astrocyte biology [42,43,44,45,46]. For example, flavonoids are known to protect the astrocytes, improve their functions, and enhance their productions [42,43,44,45,46]. Epicatechin has been reported to stimulate the antioxidant response element activity and glutathione production in mouse cortical astrocytes but not in neurons, and protect the cultured astrocytes against hemoglobin toxicity by activating nuclear factor (erythroid-derived 2)-like 2 and inhibiting the activator protein 1 [42,44]. Catechin has shown to increase the activity of superoxide dismutase, the endogenous antioxidant enzyme, in rat brain astrocytes [43]. A recent study revealed that several flavonoids such as calycosin, isorhamnetin, luteolin, and genistein enhanced the synthesis and secretion of neurotrophic factors in primary rat astrocytes via estrogen receptor [46]. However, in-depth studies are needed to fully understand how flavone derivatives regulate astrocyte function and promote astrocytogenesis especially in NSC by PD98059, compound **2,** and **3**. Like our results, flavonoids have been known to regulate NSC fate [47,48]. It has been known that epigallocatechin-3-gallate induces proliferation and promotes neurogenesis of mouse cochlear NSCs [47]. However, in-depth studies are needed to fully understand how flavonoids and flavone derivatives can regulate NSC fate.

### 3.3. 4′-Methoxylation (Methoxylation at R3 Position) Is Essential for the Induction of Neurogenesis

Immunocytochemistry analysis was performed to assess the effect of flavone derivatives on the neuronal differentiation of NSCs. To identify the differentiated neurons, TuJ1, the antibodies against βIII Tubulin were used. Among the flavone derivatives examined, only compound **6** significantly increased the differentiation of NSCs into TuJ1-positive neurons as compared with the control (Figure 2). In contrast, its 4′-demethoxylated analogs (R_3_ position) showed either no effect (PD98059, compound **4**, and **5**) or the repression of neurogenesis (compound **2**, and **3**; Figure 2). The effects of flavone derivatives on neurogenesis were further confirmed by real-time RT-PCR and western blot analysis. Consistent with the immunocytochemistry data, compound **6** increased the levels of mRNA and protein of βIII Tubulin, whereas compound **2** and **3** repressed them (Figure 3B,E,F, and Figure 4). 

The results suggested that compound **6** and its 4′-demethoxylated analogs (R_3_ position, compound **2,** and **3**) have opposite effects on neuronal differentiation in NSCs and thus elucidated the importance of the methoxy group at 4′-position (R_3_ position) in neurogenesis. Interestingly, Fatokun and coworkers reported that among the 27 flavonoids they tested, 4′-methoxy-flavone and 3′, 4′-dimethoxy-flavone (compound **6**) protect the rat cortical neurons against *N*-methyl-d-aspartate-induced cell death, and suggested that methoxylation at the 4′ position of the flavone structure is required for the activity [49]. In addition, neuroprotective and neurogenic effects of some other flavonoids have been reported [28,29,30,31,32,33,34,35]. Chroman-like cyclic prenylflavonoids promoted neuronal differentiation and neurite outgrowth in mouse embryonic forebrain-derived neural precursors and protected PC12 cells from cobalt chloride-induced cell death [30]. Wogonin has been reported to induce neurogenesis in hippocampal progenitor HiB5 cells and neurite outgrowth in rat cortical NSCs [29]. Apigenin and related compounds are reported to stimulate neurogenesis of adult rat hippocampal-derived NSCs and improve learning and memory performance in the Morris water navigation task [32]. It suggests that diverse biological activities of flavonoids and flavone derivatives depend on their chemical structure.

### 3.4. MEK Inhibition is not Required for Flavone-Induced Differentiation of NSCs

**Figure 5:**
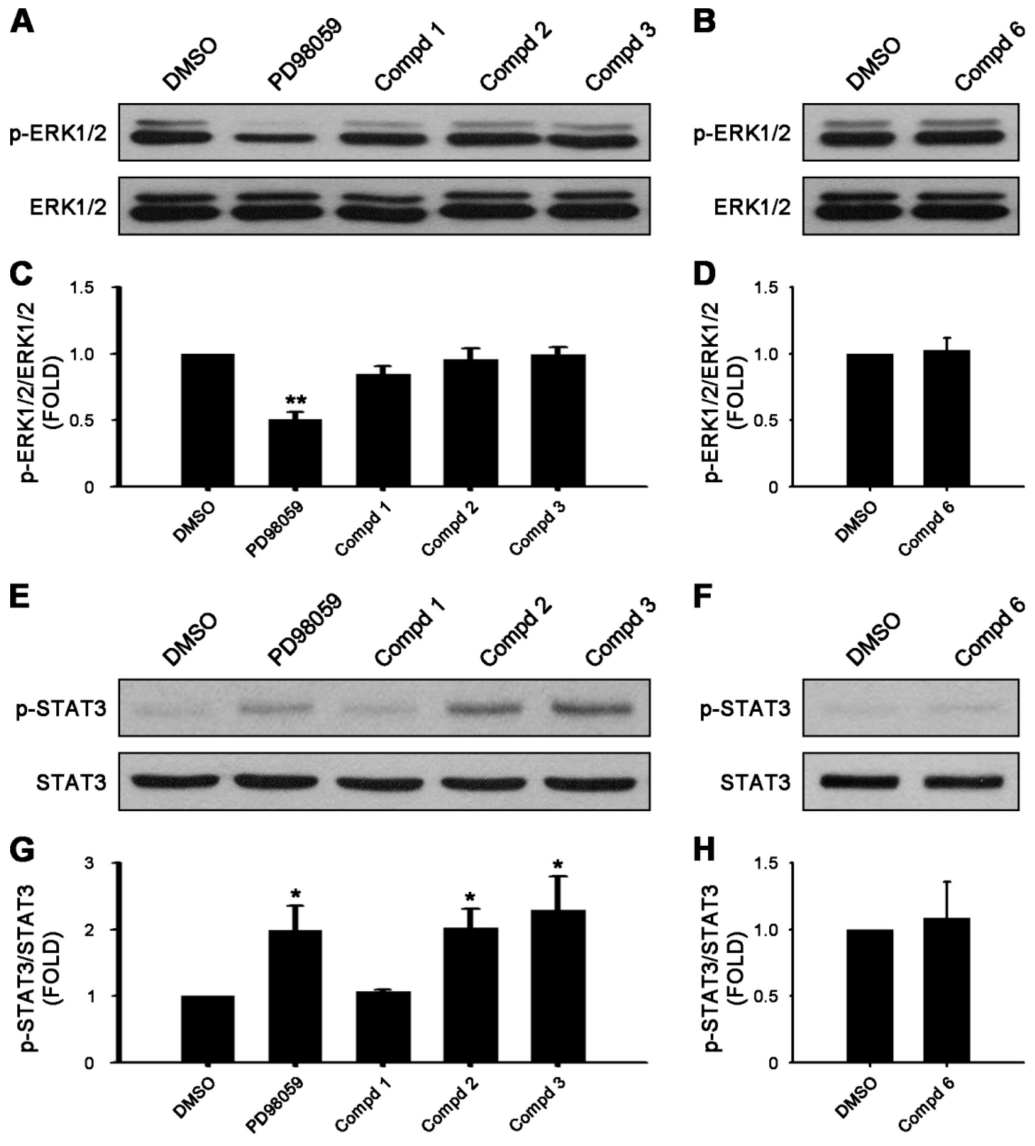
PD98059, compound **2,** and **3** induced astrocytogenesis via STAT3 activation. (**A–F**) NSC was exposed to the indicated treatment (**A–D**) for 1 h or (**E**,**F**) for 4 days, and immunoblots were performed using p-ERK1/2 (Thr202/Thr204), total ERK1/2, p-STAT3 (Tyr705), and total STAT3. Shown is a representative immunoblot and quantification of (A-D) three or (E-H) four independent experiments. * *p* < 0.05, ** *p* < 0.01 (Student’s *t*-test).

PD98059, which induces astrocytogenesis, is a flavone derivative that is a widely used MEK inhibitor that inhibits phosphorylation of the MEK substrates ERK1/2 [41,50]. To investigate the mechanisms of action of the flavone derivatives in neurogenesis or astrocytogenesis, we examined the effects of flavone derivatives on ERK1/2 phosphorylation. Western blot analysis showed that PD98059 suppressed ERK1/2 phosphorylation as expected [41], wherein the compounds **1**–**3** and **6** did not produce any significant changes (Figure 5A–D). These findings indicated that the induction of astrocytogenesis by compounds **2** and **3** and neurogenesis by compound **6** were not associated with MEK inhibition and that the flavone derivatives might be regulating the fate of NSC through a mechanism unrelated to MEK inhibition. The present study also revealed that the astrocytogenesis inducing effects of the flavone derivatives correlated with the chemical structure, the 3′-methoxy, non-bulky group at C2′ (R1) and C4′ (R3) of flavone but not with MEK inhibition. 

### 3.5. STAT3 Activation Mediates the Flavone Derivatives-Induced Astrocytogenesis

The JAK-STAT signaling pathway has been demonstrated as a mechanism controlling the astrocytic differentiation of NSCs and is a critical part of the astrogliogenic machinery [51,52,53,54,55]. Therefore, to investigate the possible mechanism of astrocytogenesis, the effects of flavone derivatives on STAT3 phosphorylation were examined. As shown in Figure 5E–H, astrocytogenesis-inducing flavone derivatives (PD98059, compounds **2,** and **3**) induced STAT3 activation, whereas none-astrocytogenic flavone derivatives (compounds **1,** and **6**) did not exert such an effect. These data reinforced the hypothesis that astrocytic differentiation by flavone derivatives is mediated via activation of STAT3.

JAK-STAT signaling pathway is a critical part of the astrogliogenic machinery [51,52,53,54,55]. Several reports, including a study from our group, have demonstrated that small molecules triggering astrocytogenesis required pre-activation of JAK-STAT pathway [9,56,57]. In the current study, we found that compound 2 increased cytokines such as *bmp2* and *il-6* transcripts and compound **3** induces **bmp2** mRNA expression (data not shown). It has been shown that astrocytogenesis inducing chemicals, such as benzothiazole derivative promoted astrocytogenesis of rat NSC via activation of STAT3 through increasing cytokines [9]. Similarly, plumbagin, a naturally occurring lipophilic phytochemicals, promoted the generation of astrocytes from rat spinal cord neural progenitors through JAK-STAT3 signaling [56]. AICAR, an adenosine analog, increased the astrocytic differentiation of immortalized neural stem cell line C17.2 and primary NSCs derived from E14 rat embryonic cortex and also activated the STAT3 phosphorylation [57]. In the current study, we observed that the flavone derivatives vary in their ability to activate this signaling pathway, and the compounds (PD98059, compound **2**, and **3**) phosphorylating STAT-3, increased astrocytogenesis but those lacking the activity did not increase astrocytogenesis. These data suggested that STAT-3 phosphorylation is indispensable for astrocytogenesis. 

## 4. Conclusions

The present study revealed that the flavone derivatives with methoxy group at 3′-position (R_2_ position) and non-bulky group at 2′- (R_1_ position) and 4′-position (R_3_ position) possessed astrogliogenic properties. Interestingly, the methoxy group at 4′-position (R_3_ position) was identified to play an essential role in neurogenic activities of the flavone derivatives. Our SAR results provided insights for the development of new flavone derivatives with improved NSC fate regulation. The present study reinforced the conclusions of previous studies revealing that the astrocytogenesis is being regulated by the structure-activity relationship of flavone derivatives, specifically through STAT3 phosphorylation but not by MEK inhibition. However, in-depth analysis is required to fully elucidate the underlying mechanism of how the flavone derivatives promote astrocytogenesis in NSC.

## Figures and Tables

**Table 1:**
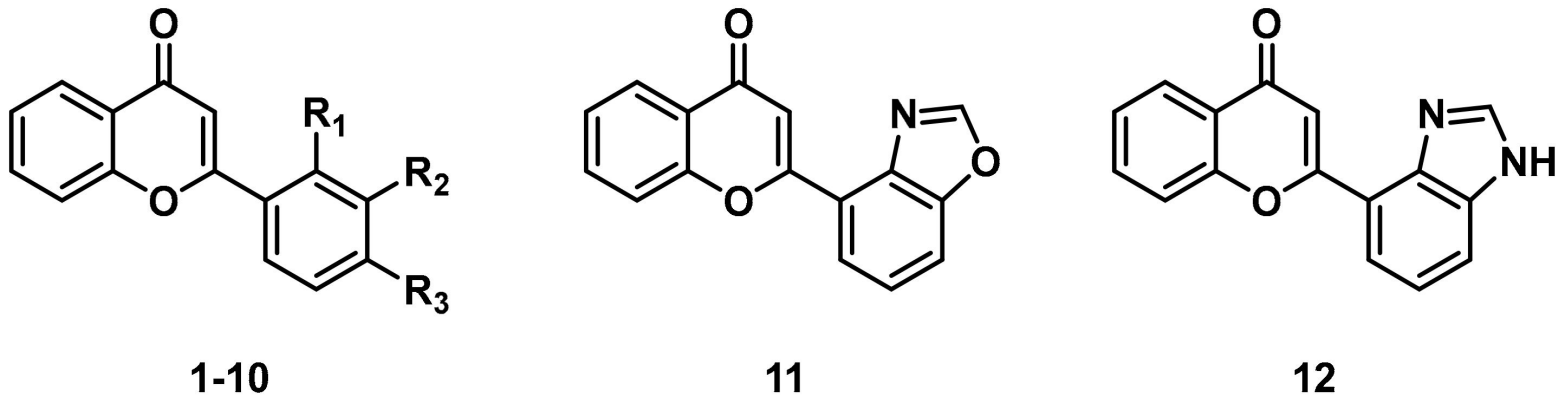
The structures of flavone and its derivatives used in this study.

	R_1_	R_2_	R_3_
Flavone	H	H	H
PD98059	NH_2_	OCH_3_	H
1	NH_2_	H	H
2	H	OCH_3_	H
3	OH	OCH_3_	H
4	OCH_3_	OCH_3_	H
5	NHCOCH_2_Ph	OCH_3_	H
6	H	OCH_3_	OCH_3_
7	NH_2_	OCH_2_Ph	H
8	H	OCH_2_Ph	H
9	NHCOCH_3_	H	H
10	NHCOCH_2_Ph	H	H
